# Resistin-like molecule alpha1 (Fizz1) recruits lung dendritic cells without causing pulmonary fibrosis

**DOI:** 10.1186/1465-9921-13-51

**Published:** 2012-06-22

**Authors:** Satish K Madala, Ramakrishna Edukulla, Katy R Davis, Stephanie Schmidt, Cynthia Davidson, Joseph A Kitzmiller, William D Hardie, Thomas R Korfhagen

**Affiliations:** 1 Division of Pulmonary Medicine, Cincinnati, OH, USA; 2 Perinatal Institute, Cincinnati Children’s Hospital Medical Center, Cincinnati, OH, USA

**Keywords:** Pulmonary fibrosis, Bleomycin, Silicosis, Fizz1, Retnla

## Abstract

**Background:**

Resistin-like molecule alpha or found in inflammatory zone protein (Fizz1) is increased in pulmonary epithelial cells and also in limited amounts by other lung cells during various lung injuries and fibrosis. However, the direct role of Fizz1 produced in the pulmonary epithelium has not been determined.

**Methods:**

Fizz1 Transgenic mice (CCSP/Fizz1) were generated that overexpress Fizz1 in the lung epithelium under the control of a doxycycline (Dox) inducible lung epithelial cell specific promoter Scgb1a1 (Clara cell secretory protein, CCSP). Histology and FACS analysis of lung cells were used to identify the direct effects of Fizz1 in the transgenic mice (Dox treated) when compared with control (CCSP/-) mice. Intratracheal bleomycin sulfate or silica in saline and saline alone were used to study the role of Fizz1 during bleomycin- and silica-induced pulmonary fibrosis in CCSP/Fizz1 and CCSP/- mice. Weight change, pulmonary inflammation, and fibrosis were assessed 10 days post bleomycin or 28 days post silica challenge.

**Results:**

When CCSP/Fizz1 mice were fed Dox food, elevated Fizz1 protein was detected in lung homogenates by western blot. Lungs of mice in which Fizz1 was induced in the epithelium contained increased lung cells staining for CD11c and F4/80 by FACS analysis consistent with increased dendritic cells however, no changes were observed in the percentage of interstitial macrophages compared to CCSP/- controls. No significant changes were found in the lung histology of CCSP/Fizz1 mice after up to 8 weeks of overexpression compared to CCSP/- controls. Overexpression of Fizz1 prior to challenge or following challenge with bleomycin or silica did not significantly alter airway inflammation or fibrosis compared to control mice.

**Conclusions:**

The current study demonstrates that epithelial cell derived Fizz1 is sufficient to increase the bone-marrow derived dendritic cells in the lungs, but it is not sufficient to cause lung fibrosis or alter chemical or particle-induced fibrosis.

## Background

Lung remodeling in the distal airspace and parenchyma is characterized by excessive extracellular matrix deposition and accumulation of apoptosis-resistant and collagen producing myofibroblasts. Currently there are no effective therapeutic treatments available for pulmonary fibrosis highlighting the importance of identifying targetable cells, fibrogenic pathways and molecules to design therapeutic approaches and to use as biomarkers of disease progression. Multiple rodent models have been established that recapitulate mechanisms leading to pulmonary fibrosis and identify cells, pathways and growth factors mediating pulmonary fibrosis [1-6]. Resistin-like molecules or found in inflammatory zone (FIZZ) are a family of cysteine-rich secreted small proteins which have been shown to play an important role in the cell differentiation implicated in the pathophysiology of chronic diseases including obesity, type 2 diabetes, helminth infections and forms of pulmonary fibrosis [7-12]. The family consists of 4 members: Relm-α/Fizz1, Relm-β/Fizz2, Resistin/Fizz3, and Relm-γ/Fizz4. In the lung, Fizz1 is induced in environments with Th2-cytokine preponderance and induction of Fizz1 occurs through IL13/IL4-driven STAT 6 activation. In hypoxic injury, Fizz1 is induced independent of IL4 [8]. The expression pattern of Fizz1 resembles resistin suggesting functional overlap between mouse derived Fizz1 and human resistin [13]. *In vitro* studies demonstrate that Fizz1 activates type I collagen expression in fibroblasts *via* a notch1-dependent pathway [10]. In addition, Fizz1 transforms fibroblasts to collagen producing myofibroblasts and induces anti-apoptotic responses in myofibroblasts contributing to excess myofibroblast accumulation and production of extracellular matrix proteins in the lung [10].

Previous studies provide conflicting evidence regarding the *in vivo* involvement of Fizz1 in promoting forms of pulmonary fibrosis [7-10,14]. Fizz1 overexpression using adenoviral gene transfer or hypoxia increased the recruitment of bone marrow derived cells (BMD) to the lung and increased vascular remodeling with fibrotic changes localized to pulmonary arteries [8,15]. In a mouse model of hypoxia-induced pulmonary hypertension, the recruitment of BMD cells was associated with a concomitant increase in Fizz1 levels in the lung. However, Fizz1 knockout mice challenged with the parasitic eggs of *S. mansoni* eggs displayed heightened pulmonary fibrosis associated with increases in CD4+ Th2 cell derived IL-4 and IL-13 [7,9] suggesting that Fizz1 is a negative regulator of pro-fibrotic Th2 cytokines induced by parasitic infections.

Transforming growth factor-α (TGFα) is a ligand for the epidermal growth factor receptor (EGFR) and transgenic mice conditionally expressing TGFα specifically in the lung epithelium develop progressive fibrosis accompanied with cachexia, changes in lung mechanics and secondary pulmonary hypertension [16,17]. Microarray analysis of whole lung homogenates in the TGFα mice demonstrates increases in Fizz1 transcripts following the induction of the transgene [16]. Fizz1 was originally reported to be induced primarily in lung epithelial cells in Ova [18] and Aspergillus models [19] and in a hypoxia model [8]. Fizz1 is also induced in macrophages in asthma models and eosinophils in asthma and helminth infection models [7,19]. More recently Fizz1 has been reported in alternatively activated macrophages [20]. Recent studies using Fizz1 reporter mice confirm that lung epithelial cells are a major source for Fizz1 in the lung [7]. As the direct role of Fizz1 in the pathogenesis of lung fibrosis remains elusive and the lung epithelial cells appear to be key producers of Fizz1, we generated transgenic mice which conditionally expresses Fizz1 under the control of a doxycycline (Dox) inducible lung epithelial cell specific promoter Scgb1a1 (Clara cell secretory protein, CCSP). This promoter has been shown to lead to transgene expression in the bronchial, bronchiolar epithelium and to a lesser extent in alveolar epithelium [21,22]. This pattern of gene expression mimics the cellular sites reported for Fizz1 expression in the lung [7,18,19]. Transgenic mice constitutively expressing Fizz1 in the pulmonary epithelium were used to determine effects on cellular influx in the lung, lung remodeling, and experimental models of pulmonary fibrosis using bleomycin or silica.

## Methods

### Generation of transgenic mice

**Figure 1:**
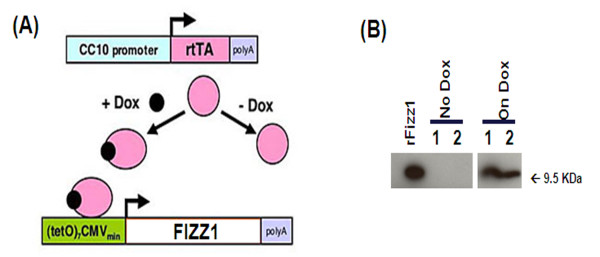
**Generation of CCSP/Fizz1 transgenic mice. (A)** Gene targeting strategy for generating conditional CCSP/Fizz1 transgenic mice. TetO-CMV Fizz1^+/+^ mice were generated by cloning full length Fizz1 cDNA from mouse lung RNA and inserting into the (TetO)_7_-CMV promoter. CCSP/Fizz1 transgenic mice were produced by mating single transgenic CCSP/- mice with (TetO)_7_-CMV Fizz1^+/+^ mice. To induce Fizz1 expression in bitransgenic mice were administered doxycycline (Dox) as described in methods. **(B)** Western blot shows Fizz1 overexpression in the lungs of CCSP/Fizz1 mice on Dox for two weeks. rFizz1 is a lane containing purified Fizz1 (Peprotech) loaded as a positive control. Lanes 1, 2 are duplicate samples. The expressed Fizz1 in Dox treated transgenic mice migrated identically to the Fizz1 marker compared to CCSP/Fizz1 mice without Dox treatment. The relative intensity of the bands in the On Dox lanes was increased about 14.1 fold.

Transgenic mice were generated by cloning full length Fizz1 cDNA from mouse lung RNA and inserting into the (TetO)_7_-CMV promoter. The (TetO)_7_-CMV Fizz1 transgene consists of seven copies of tet operator DNA binding sequence linked to a minimal CMV promoter, the mouse Fizz1 cDNA, and SV40 polyadenylation signal. The construct was injected into mouse oocytes generating (TetO)_7_-CMV Fizz1^+/+^ mice. All mice were derived from the FVB/NJ inbred strain. Animals were housed under specific pathogen-free conditions and handled in accordance with protocols approved by the Institutional Animal Care and Use Committee of the Children's Hospital Research Foundation and the University of Cincinnati Medical Center. Bitransgenic Fizz1 transgenic mice (abbreviated as CCSP/Fizz1) were produced by mating Single transgenic Clara Cell Specific Protein-rtTA^+/−^ mice (abbreviated as CCSP/-) with (TetO)_7_-CMV Fizz1^+/+^ mice. To induce Fizz1 expression bitransgenic mice were administered doxycycline (Dox) food (62.5 mg/kg). The fold increase in detectable Fizz1 was determined by comparing pixels from the off Dox and On Dox lanes in Figure 1 using a phosphorimager and ImageQuant software [23].

#### PCR Genotyping

Transgenic mice were identified using PCR primers specific for each transgene as follows: 5' primer in the rat CCSP promoter, 5' ACT GCC CAT TGC CCA AAC AC-3'; 3' primer in rtTA coding sequence, 5'-AAA ATC TTG CCA GCT TTC CCC-3'. Mice were identified for (TetO)7-CMV Fizz1 transgene with PCR sense primer, 5' TGA TGG TCC CAG TGA ATA CTG ATG-3'; anti-sense primer, 5' GGT CCA TGG TGA TAC AAG GGA CAT-3'. Amplification of PCR products for CCSP and Fizz1 were performed by denaturation at 94°C for 5 minutes and then 30 cycles of amplification at 94°C for 30 seconds, 57°C for 30 seconds and 72°C for 30 seconds, followed by 7 minutes extension at 72°C.

### Western blot

Proteins in the total lung homogenates (50 μg) from the CCSP/Fizz1 mice on Dox or off Dox for 2 weeks were separated using SDS-PAGE and proteins transferred to PVDF membrane as described earlier [23]. Fizz1 protein was detected using a rabbit polyclonal anti-mouse Fizz1 antibody (Peprotech, Rocky Hill, NJ, USA) and HRP-conjugated anti-rabbit secondary antibody. Recombinant Fizz1 protein was used as a positive control and signal developed using a chemiluminescent kit.

### Mouse model of bleomycin- and silica- induced fibrosis

Bleomycin-induced pulmonary fibrosis was performed as described earlier with few modifications [24]. In brief, CCSP/- and CCSP/Fizz1 mice were kept on Dox for two weeks to establish Fizz1-induced changes in the lung. On day 14, mice were anaesthetized with a xylazine and ketamine cocktail and given 0.15 U bleomycin sulfate (EMD) in saline or saline alone *via* the I.T. route. On day 10 post bleomycin, mice were sacrificed and the lungs were collected for biochemical and histological analysis.

For silica-induced pulmonary fibrosis, mice on Dox were anaesthetized with a xylazine and ketamine cocktail and given 3 mg of 5 micron size sterile silica (US silica, Beverly Springs, WV, USA) per mouse in 50 μl saline or saline alone *via* the I.T. route. On day 28, mice were sacrificed and the lungs were collected for biochemical and histological analysis.

### Lung histology and hydroxyproline measurements

Lungs were inflation fixed using 4% paraformaldehyde and stained with hematoxylin and eosin (H&E) and masson trichrome kit as described earlier [23].

Fibrosis in the lungs was quantified using a modified assay for determination of hydroxyproline in tissues [25]. In brief, the left lobe of lung was weighed and hydrolyzed in 4 ml of 6 N HCl overnight at 110°C. Hydrolyzed samples were neutralized with 1 N NAOH. For colorization, chloramine-T (0.05 M) and the aldehyde-perchloric acid reagent were added and samples placed in hot water bath at 60°C for 25 min. The standard hydroxyl-L-proline (EMD) solutions prepared and treated identically to the samples to read at 550 nm for samples and standards. Sample concentrations were determined from the standard curve.

### Quantification of broncho-alveolar lavage cells

Mice challenged with bleomycin or silica were euthanized with sodium pentobarbital (65 mg/kg) and lungs were lavaged sequentially with 0.7 and 0.5 ml aliquots of PBS, pooled and used to determine total cell counts and for cytospin preparations to determine macrophage cell counts. Cells were isolated from the broncho-alveolar lavage fluid (BALF) by centrifugation at 1250 rpm-5 minutes and the cell pellet resuspended in 1 ml of PBS and cells were counted using hemocytometer.

### FACS staining

Total lung cells were prepared for FACS staining by mincing the lung in a complete RPMI medium and subjected to collagenase digestion for 30 minutes at 37°C. After digestion, lung cells were filtered through 100 μm filter and counted by hemocytometer. About 1 million cells were incubated with CD16/CD32 (Fc block) and stained for surface antigens CD11b, CD11c, F4/80 and CD45. Cell populations were analyzed using FACS Calibur and Flow Jo as described earlier [26].

### RNA preparation and real-time PCR

Total RNA was extracted from lung tissue using the RNeasy Mini Kit from Qiagen (Qiagen Sciences, Valencia, CA) as described previously [26]. Primers used were: HPRT 5'-GCCCTTGACTATAATGAGTACTTCAGG-3' (forward), 5'-TTCAACTTGCGCTCATCTTAGG-3' (reverse); Fizz1 5'-CCCTCCACTGTAACGAAGACTC-3' (forward), 5'-CACACCCAGTAGCAGTCATCC-3' (reverse).

### Statistics

All data was analyzed with Prism (Version 5; GraphPad). Student *t*-test used to compare between two groups. One-way ANOVA with Tukey`s Multiple Comparison post-test is used to compare more than two experimental groups and data were considered statistically significant for P values less than 0.05.

## Results

### Generation of Fizz1 transgenic mice

Transgenic mice were generated by cloning full length Fizz1 cDNA from mouse lung RNA and inserting into the CMV-TetO promoter (TetO-Fizz1 mice). 4 different lines were generated and screened for increases in Fizz1 transcripts. Selected TetO-Fizz1 mice were bred to CCSP-rtTA activator mice to generate bitransgenic CCSP-rtTA/tetO-Fizz1 mice (CCSP/Fizz1) conditionally expressing epithelial Fizz1 protein when fed Dox containing food (Figure 1A). To demonstrate the proper translation and maturation of overexpressed Fizz1 transcripts, CCSP/Fizz1 mice were administered Dox food for 2 weeks and Fizz1 protein measured in lung homogenates by western blot. CCSP/Fizz1 mice on Dox demonstrated about 14.1 fold increases in the signal intensity at 9.5 kDa corresponding to a mature Fizz1 protein in lung homogenates over lung homogenates from mice not treated with Dox (Figure 1B). Previous studies demonstrate that target gene expression in bitransgenic mice using the reverse tetracycline transactivator (rtTA) expressed under the control of CCSP promoter is restricted to alveolar Type II cells and non-ciliated bronchial and bronchiolar epithelial cells but not in ciliated cells[27-29]. The highest-expressing line of founders was subsequently used in all studies.

### Effect of Fizz1 overexpression in the lungs

**Figure 2:**
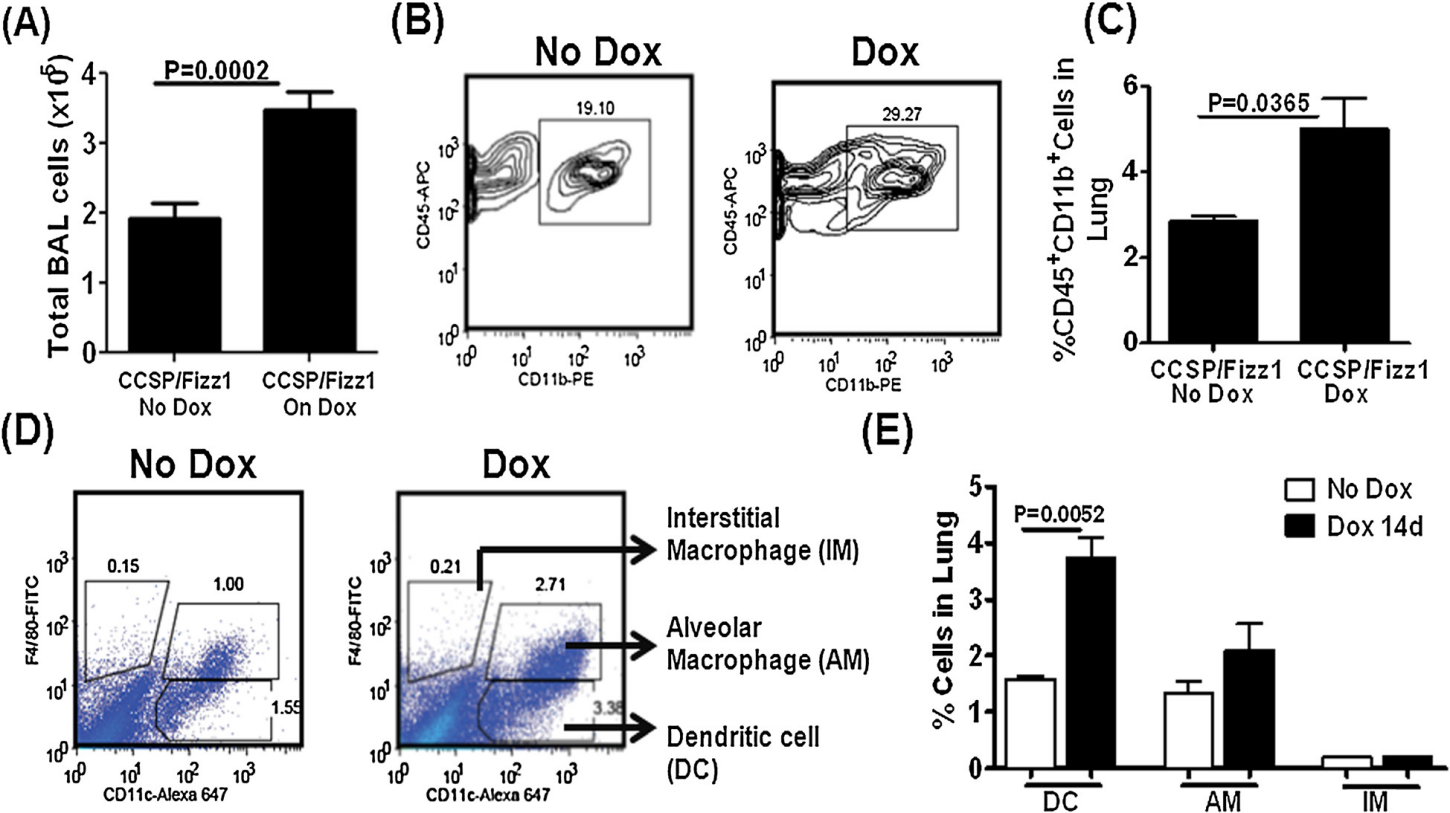
**The lung epithelial specific Fizz1 overexpression increases the percentage of dendritic cells in the lung. (A)** CCSP/Fizz1 mice were fed doxycycline food for 14 days. Total BAL cells increased with Fizz1 overexpression in CCSP/Fizz1 mice. Statistical significance is measured using unpaired student’s *t* test (n = 11/group). **(B)** CCSP/Fizz1 mice were fed doxycycline food for 14 days and total lung cells were stained with anti-CD11b and anti-CD45 antibodies. Gating strategy and representation of CD45+ and CD11b + cells in the lungs of Fizz1 overexpressing mice compared to CCSP/Fizz1 mice on no Dox. **(C)** Fizz1 overexpression has caused increase in the percentage of CD45 and CD11b double positive cells. Statistical significance is measured using unpaired student’s *t* test (n = 3/group). **(D)** CCSP/Fizz1 mice were fed doxycycline food for 14 days and total lung cells were stained with anti-CD11c and anti-F4/80 antibodies. Gating strategy and representation of DC, AM and IM in the lungs of Fizz1 overexpressing mice compared to CCSP/Fizz1 mice on no Dox. **(E)** Mice overexpressing Fizz1 demonstrate increases in the percentage of cells staining for CD 11c that do not stain for F4/80 consistent with changes in dendritic cells. Statistical significance is measured using unpaired student’s *t* test (n = 3/group). Data represent one of two independent experiments showing similar results.

To assess the direct effects of Fizz1 overexpression, CCSP/Fizz1 mice were fed Dox food for 14 days. CCSP/Fizz1 mice on Dox demonstrated increased total cell counts compared to mice without Dox (Figure 2A). There were no differences between groups in cytospin differential with 99% macrophages recovered.

Fizz1 has previously been reported to increase recruitment of bone marrow derived cells to the lung [30]. To assess if epithelial overexpression of Fizz1 altered bone-marrow-derived cell populations in the lung, total lung cells from lung homogenates of CCSP/Fizz1 mice on and off Dox were stained for markers of hematopoietic cells (CD45) and monocytes/macrophages (CD11b). CCSP/Fizz1 mice on Dox demonstrated increases in both the percentage and total number of cells positive for both CD45 and CD11b (Figure 2B and 2C). Total lung cells were also stained for CD11c and F4/80, which are markers distinguishing dendritic cells from alveolar (AM) and interstitial macrophages (IM) [31]. CCSP/Fizz1 mice on Dox demonstrated a more than two fold increase in dendritic cells with no significant changes in AM and IM (Figure 2D and 2E). Together, these experiments show that overexpression of Fizz1 in the lung resulted in an increased accumulation of bone-marrow-derived dendritic cells, consistent with previous reports [8,30]. We measured the transcripts for chemokines CCL3 and CCL19, which are known to be involved in the recruitment of DCs to the lung. CCL3 and CCL19 transcript levels are not changed in Fizz1 overexpressing mice compared to control mice at day 5 on Dox (Additional file 1: Figure S 1). Therefore, mechanisms involved in the accumulation of DCs in Fizz1 overexpressing mice could be direct, due to Fizz1, or alternatively other unknown modulators could be induced which would make the effects of Fizz1 indirect.

**Figure 3:**
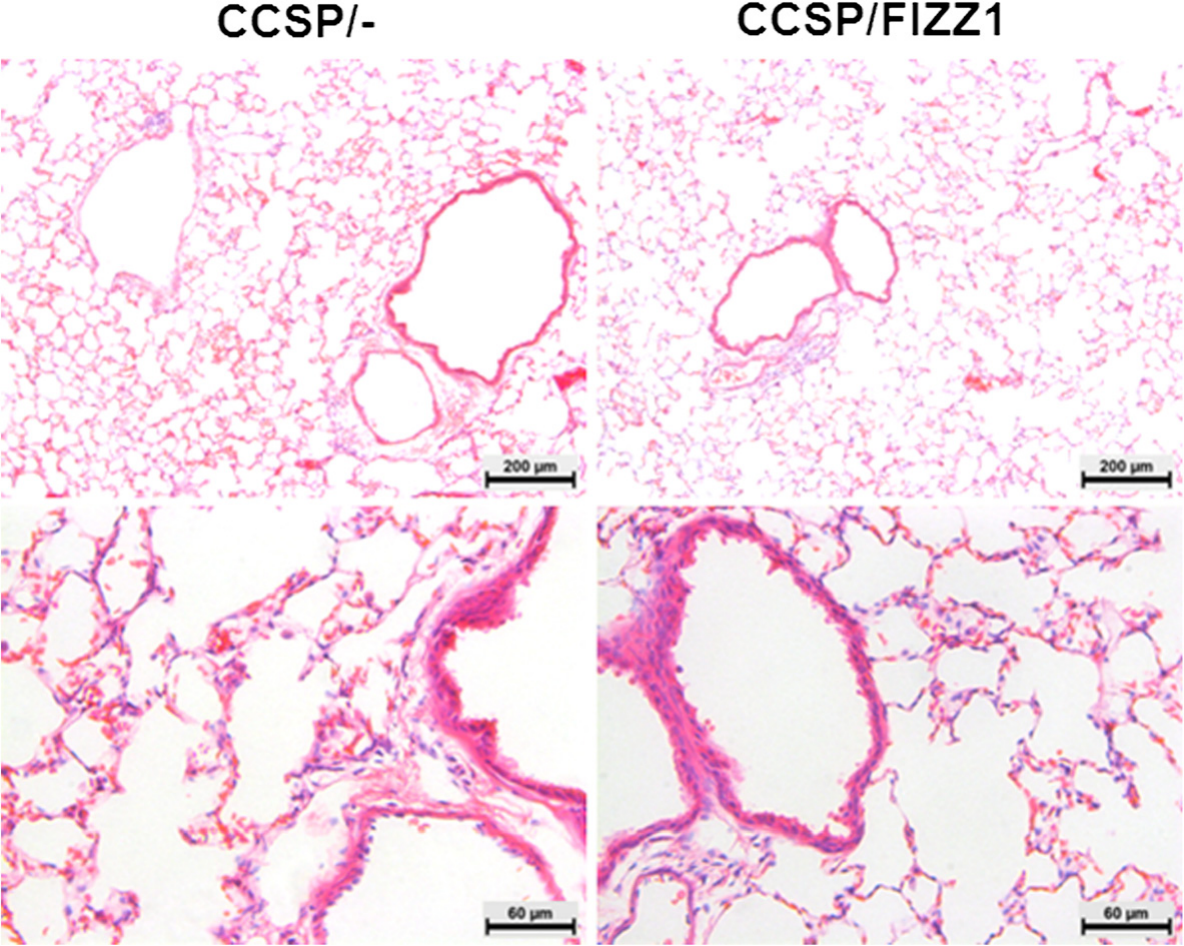
**The effect of Fizz1 overexpression on the lung histology.** H&E stained lung sections from CCSP/- and CCSP/Fizz1 mice on Dox for 8 weeks at 10X (top) & 20X (bottom) magnification. Histological examination reveals no detectable changes in the morphology of airways, blood vessels and also inflammatory cells in the lung tissues of CCSP/- and CCSP/Fizz1 mice on Dox for 8 weeks.

To assess the direct effects of Fizz1 overexpression on lung remodeling, lung histology was assessed in CCSP/Fizz1 mice at selected intervals up to 8 weeks of continuous expression. There were no detectable changes in collagen deposition, myofibroblasts accumulation or vascular remodeling between CCSP/Fizz1 mice on Dox compared to controls (Figures 3). Together, these data suggest that Fizz1 increases the migration of bone-marrow derived cells to the lung but has no direct effects on pulmonary remodeling in normal mice without known inflammatory injuries.

### Effect of Fizz1 overexpression on bleomycin-induced inflammation and fibrosis

**Figure 4:**
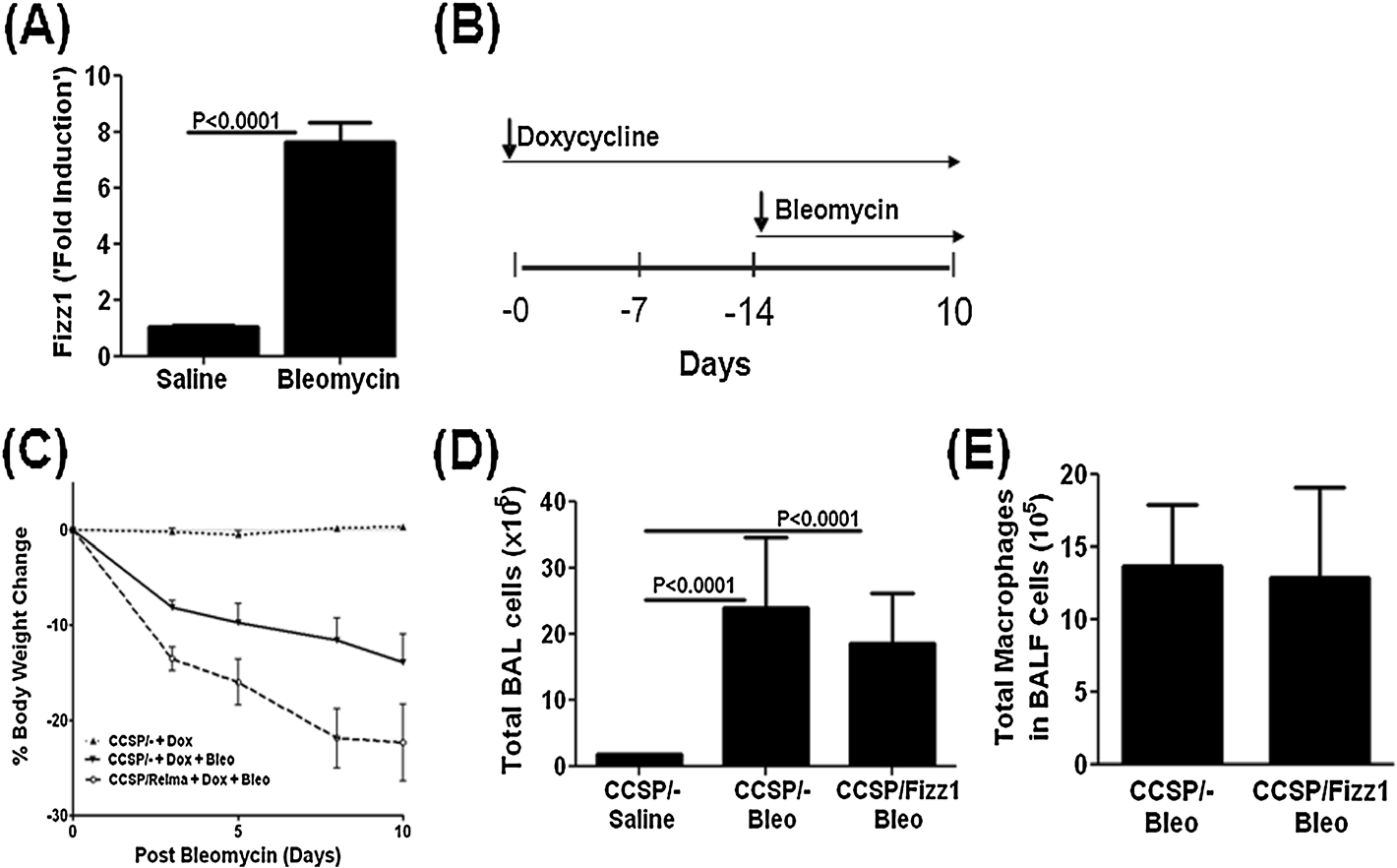
**Fizz1 overexpression in the lung has no effect on bleomycin-induced inflammation. (A)** CCSP/- mice lungs were exposed to saline or bleomycin *via* the I.T. route. Fizz1 transcripts were measured using RT-PCR at day10 post-bleomycin. Statistical significance is measured using unpaired student’s *t* test (n = 5/group). **(B)** Schematic representation of Fizz1 induction by feeding doxycycline food and administration of 0.15U of bleomycin *via* the I.T. route. **(C)** CCSP/Fizz1 mice exposed to bleomcyin demonstrate accelerated weight loss compared with CCSP/- controls receiving bleomycin. Data shown are mean ± SEM (n = 7-8/group). **(D)** Total BALF cells quantified at day10 post bleomycin or saline. Data shown are mean ± SEM (n = 7-8/group). Statistical significance is measured using unpaired student’s *t* test. **(E)** Absolute counts of macropahges in BALF cells were obtained from cell differentials. Data shown are mean ± SEM (n = 7-8/group). Data represent one of two independent experiments showing similar results.

**Figure 5:**
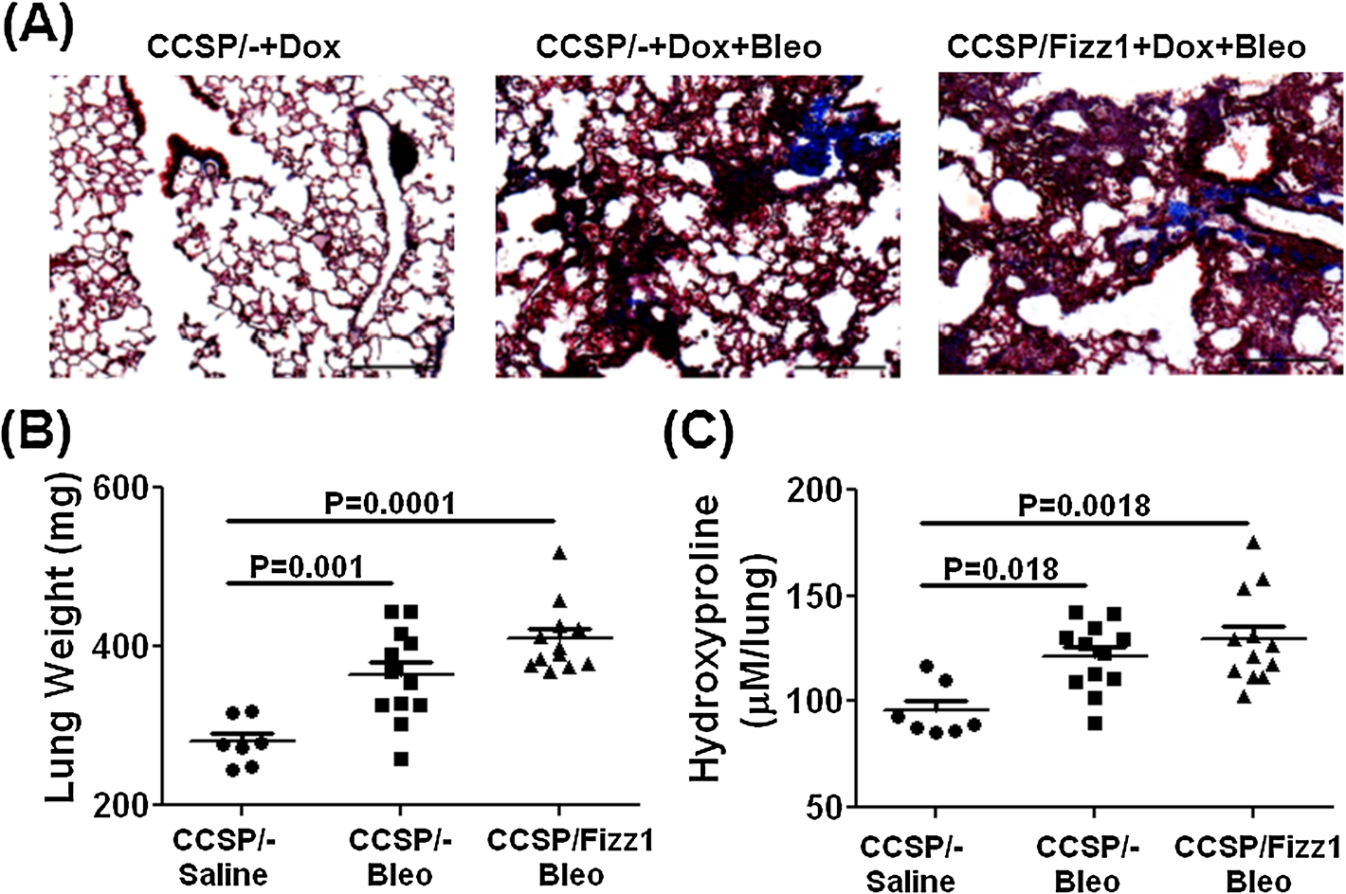
**Fizz1 overexpression has no effect on bleomycin-induced fibrosis in the lung.** CCSP/- and CCSP/Fizz1 mice were exposed to saline or bleomycin (0.15U) *via* the I.T. route, with pulmonary fibrosis assessed at day 10. **(A)** Masson Trichrome stained lung sections at 10X magnification. Scale bar 200 μm. **(B)** Total lung weights were measured in all groups at day10 post-bleomycin. **(C)** The lung collagen deposition, expressed as micromoles of hydroxyproline per lung. The above data is an average of two independent experiments with the total 7–14 mice per group. Statistical significance is measured using one way ANOVA (n = 7-14/group).

We next assessed if overexpression of lung epithelial Fizz1 altered the phenotype of inflammatory models of pulmonary fibrosis. Fizz1 transcripts in wild type mice 10 days following bleomycin injury were increased more than 6-fold from controls confirming activation of Fizz1 with bleomycin injury (Figure 4A). CCSP/Fizz1 mice were fed Dox food for 14 days to establish Fizz1-induced changes in the lung cells. Mice were challenged with intratracheal bleomycin on day 14 and continued on Dox for another 10 days before mice were euthanized (Figure 4B). CCSP/Fizz1 mice on Dox receiving bleomycin had a greater loss in body weight compared with CCSP/- control mice on Dox and bleomycin (Figure 4C). Total BAL cells were increased in both CCSP/Fizz1 and CCSP/- controls at day10 post bleomycin challenge (Figure 4D). Compared with saline treated controls there were no differences in BAL cell differentials between bleomycin-challenged groups (Figure 4E). Bleomycin-induced lung injury has been shown to cause the recruitment of CD11b^+^ DCs which may contribute to the fibrotic responses in the lung [32]. In agreement, bleomycin-induced injury has resulted in the accumulation of CD45^+^CD11b^+^ cells in the lungs of CCSP/- and CCSP/Fizz1 mice exposed to bleomycin compared to saline treated CCSP/- mice (Additional file 1: Figure S2). Fizz1 overexpression had no effect on the percentage of CD45^+^CD11b^+^ cells compared to CCSP/- mice exposed to bleomycin. Masson trichrome staining did not demonstrate differences in the degree or distribution of matrix deposition in the lungs following bleomycin in the CCSP/Fizz1 and CCSP/- mice challenged with bleomycin (Figure 5A). Total lung weight and hydroxyproline levels in the lungs were increased in the CCSP/Fizz1 and CCSP/- mice following bleomycin, but there were no differences between groups (Figure 5B and 5C). Together, these data indicate Fizz1 overexpression resulted in greater loss in body weight following bleomycin, but did not alter the inflammatory cells or the total collagen content in the lung, suggesting that Fizz1 had no effect on bleomycin-induced pulmonary fibrosis.

### Effect of Fizz1 overexpression on silica-induced inflammation and fibrosis

**Figure 6:**
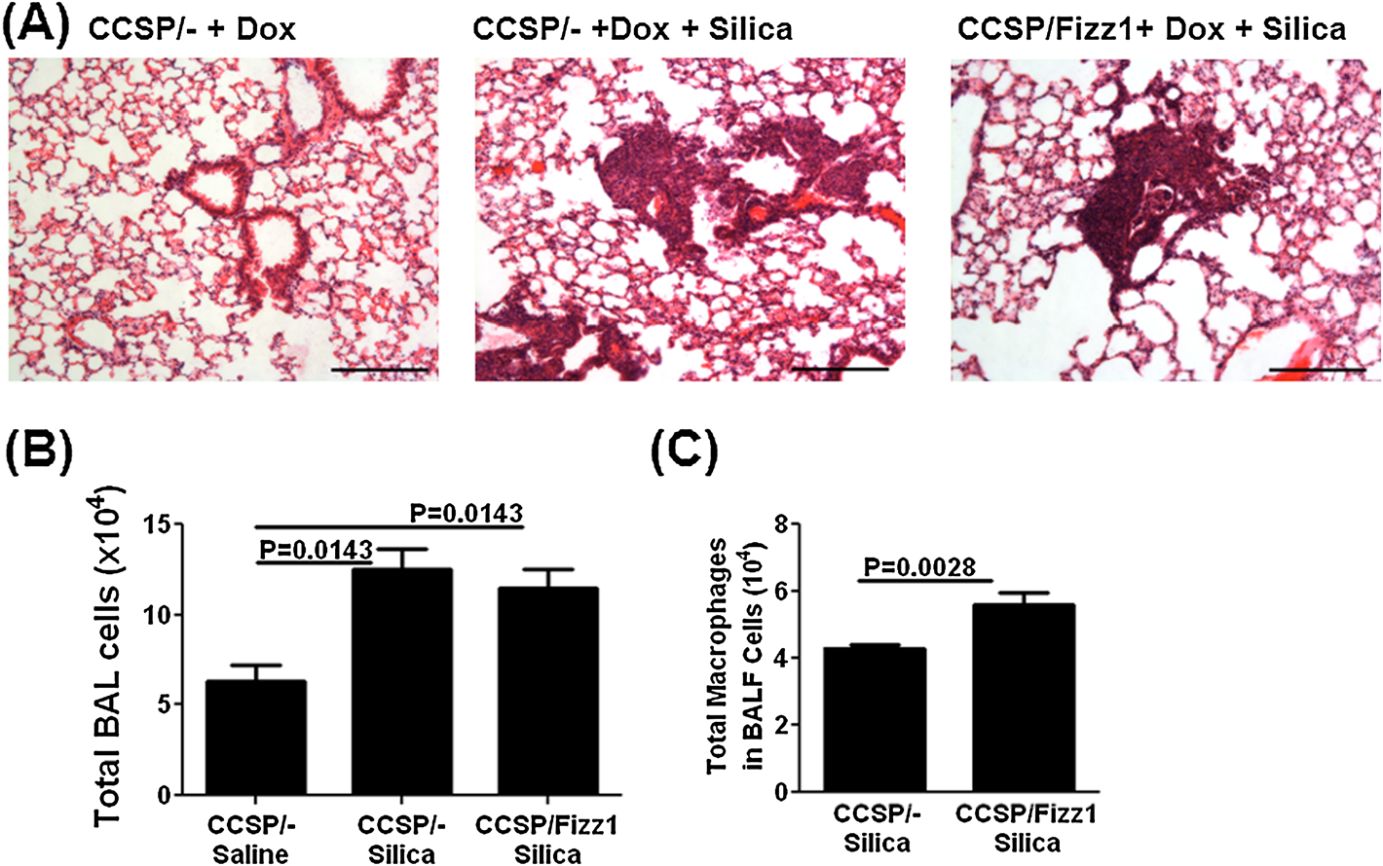
**Fizz1 overexpression has no effect on silica-induced granulomatous inflammation in the lung.** CCSP/- and CCSP/Fizz1 mice were exposed to saline or silica (3 mg/mice) *via* the I.T. route, pulmonary inflammation assessed on day 28 after silica instillation. Data represent one of two independent experiments showing similar results with five animals per group. Data shown are mean ± SEM. Statistical significance is measured using one way ANNOVA (n = 5/group). **(A)** H&E stained lung sections at 10X magnification. Scale bar 200 μm. CCSP/- and CCSP/Fizz1 mice exposed to silica develop granulomatous nodules of similar size **(B)** Total BAL cells counted at day 28 post silica or saline. **(C)** Absolute counts of macrophages in BALF cells were obtained from cell differentials.

**Figure 7:**
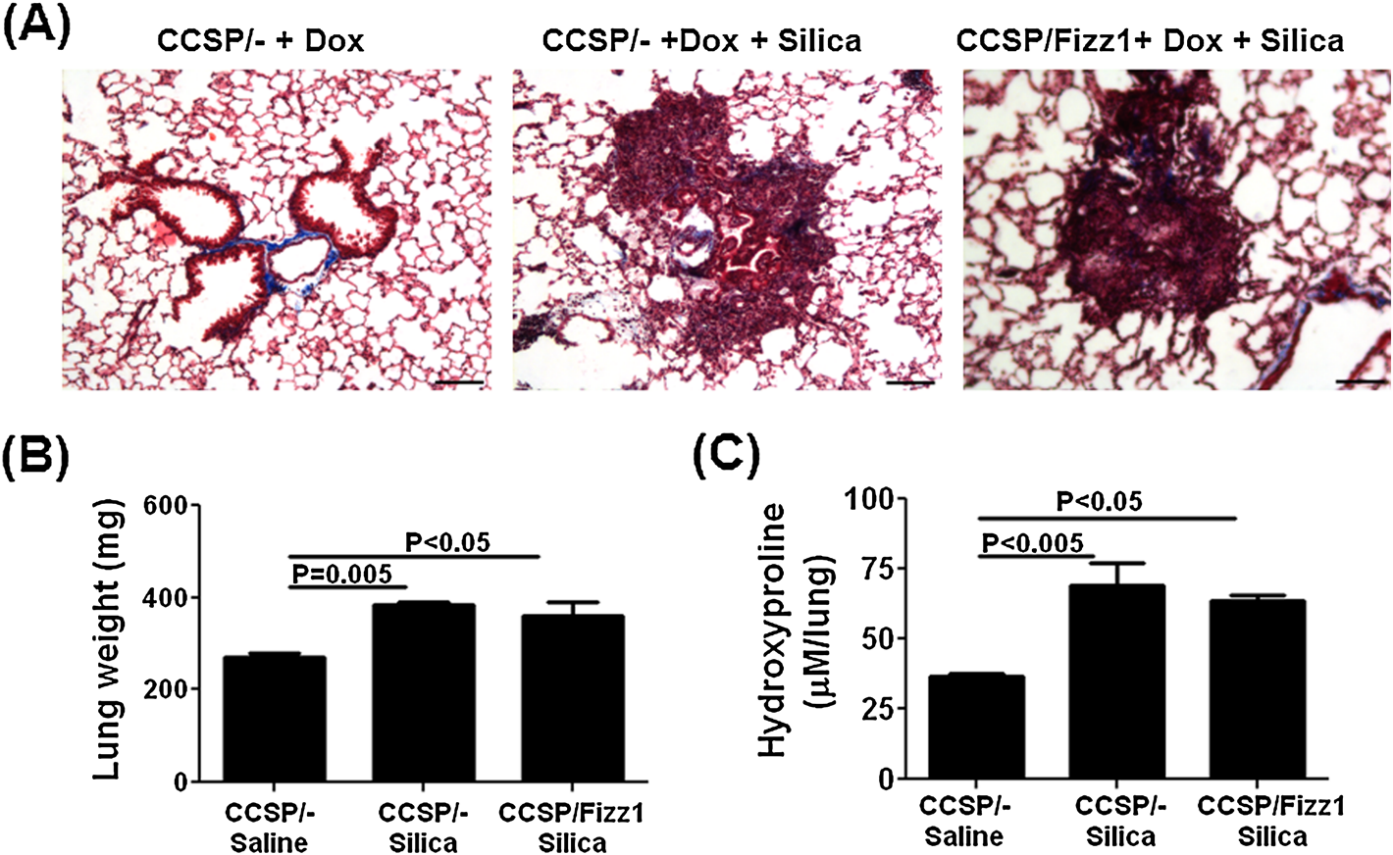
**Fizz1 overexpression has no effect on silica-induced fibrosis in the lung.** CCSP/- and CCSP/Fizz1 mice were exposed to saline or silica (3 mg/mice) *via* the I.T. route, with pulmonary fibrosis assessed on day 28 after silica instillation. One of two independent experiments is shown with five animals per group. Data shown are mean ± SEM. Statistical significance is measured using one way ANOVA . **(A)** Masson Trichrome stained lung sections at 10X magnification. CCSP/- and CCSP/Fizz1 mice exposed to silica develop granulomatous nodules of similar size. Scale bar 200 μm. **(B)** Total lung weights were measured in all groups at day 28 post-silica. CCSP/- and CCSP/Fizz1 mice exposed to silica showed increases in the lung weights and no difference with Fizz1 overexpression. **(C)** The lung collagen deposition, expressed as micromoles of hydroxyproline per lung. CCSP/- and CCSP/Fizz1 mice exposed to silica showed increases in the lung weights and no difference with Fizz1 overexpression.

Occupational exposure to crystalline silica has been shown to form silicotic nodules characterized by localized inflammation, fibroblast proliferation and excess collagen deposition [33-35]. Resident and recruited macrophages have been shown to play a pivotal role in developing silicotic nodules, and increased proportions of Fizz1 expressing macrophages are found in bronchoalveolar lavage specimens from animals and humans with silica dust exposure [35-39]. To determine whether Fizz1 overexpression altered silica-induced pulmonary inflammation and fibrosis, CCSP/Fizz1 were fed Dox food then challenged with intratracheal silica and continued on Dox for another 28 days following silica instillation. CCSP/Fizz1 and CCSP/- control mice on Dox developed granulomatous nodules largely comprising of macrophages but there were no differences in the numbers or distribution of nodules within groups (Figure 6A). Total BAL cells were increased in both CCSP/Fizz1 and CCSP/- controls but there were no differences between groups and no differences in cell differential (Figure 6B). Total macrophage numbers in BAL were increased in CCSP/Fizz1 mice compared to CCSP/- mice exposed to silica (Figure 6C). Mason’s trichrome staining did not demonstrate differences in the degree or distribution of matrix deposition in the lungs following silica in either group (Figure 7A). Total lung weight and hydroxyproline levels in the lungs were increased in the CCSP/Fizz1 and CCSP/- controls following silica, but there were no differences between groups (Figure 7B and 7C). Together, these data indicate that Fizz1 overexpression did not alter inflammation or the total collagen content in the lungs of silica-exposed mice.

## Discussion

The current study demonstrates that constitutive overexpression of Fizz1 in lung epithelial cells increases the percentage of bone marrow derived hematopoietic cells in the lung. Our findings support earlier work where Fizz1 was overexpressed in the lung using a recombinant adeno-associated viral (AAV) vector, and bone marrow derived cells of hematopoietic lineage were recruited to the smooth muscle layer of pulmonary vessels [30]. In the current study we further characterized the changes in the lung cell population with Fizz1 overexpression using FACS analysis and demonstrated that Fizz1 induced recruitment was associated with a higher percentage of cells staining for both CD11c and F4/80, which are markers associated with dendritic cells. This is the first study to demonstrate a direct role for Fizz1 produced in lung epithelial cells *in vivo* in the recruitment of dendritic cells into the lung and further supports a role for the Fizz family as a chemoattractant for bone marrow derived cells. Recently, Fizz2 has also been shown to directly cause chemoattraction of bone-marrow-derived dendritic cells, and lung recruitment of bone-marrow-derived cells including dendritic cells was impaired in Fizz2 knockout mice [40].

Our study demonstrated that epithelial overexpression of Fizz1 without a concomitant lung injury was not associated with changes in lung architecture or lung fibrosis. The role of Fizz1 *in vivo* in altering pulmonary fibrosis is unclear. Fizz1 overexpression with adenoviral vectors increased thickening and collagen surrounding pulmonary arteries [30]. However, Fizz1 knockout mice challenged with the parasitic eggs of *S. Mansoni* eggs displayed heightened pulmonary fibrosis suggesting that Fizz1 is a negative regulator of parasite induced pro-fibrotic Th2 cytokines and in this model Fizz1 is anti-fibrotic [7]. Another recent study using Fizz1 KO mice demonstrates that Fizz1 was largely dispensable during *Aspergilus fumigatous* allergen-induced production of Th2 cytokines, eosinophilia and airway remodeling [19].

The migration of myeloid cells from the bone marrow to the lung has been shown to regulate inflammation and the pathogen burden in the lung. Data over the past decades also supports a role for bone marrow derived fibrocytes in contributing to the cellular accumulation of mesenchymal cells and extracellular matrix [41]. Although, myeloid cells have been shown to migrate into the lung, the secretory mediators involved in the migration and maturation to different cell subsets, along with their functional role remains an area of active investigation. The present study does not establish whether Fizz1 chemoattraction of dendritic cells is due to Fizz1 alone or Fizz1 regulation or interaction with other chemoattractant molecules. The myeloid cell subsets that populate in the lung include dendritic cells, alveolar macrophages and interstitial macrophages. Notably, findings of this study demonstrate that Fizz1 overexpression increased dendritic cells in the lung without altering lung architecture. Dendritic cells are critical members of adaptive immunity in the lung involved in antigen processing and mediating immunity responses. Lung dendritic cells have been shown to accumulate in human fibrotic lung disease and bleomycin-induced fibrosis, but have not been shown to independently and directly cause lung remodeling. The present study shows that Fizz1 mediated attraction of dendritic cells was not adequate to influence lung fibrosis. Fizz1 overexpression has no effect on CD45 + CD11b + cells in the lung during bleomycin-induced injury. Future studies are warranted to identify the roles of Fizz1-recruited dendritic cells in immunity against bacterial and viral pathogens and also infections associated injury and remodeling in the lung.

As we were not able to demonstrate direct remodeling from Fizz1 overexpression, we examined if Fizz1 overexpression altered fibrosis in known inflammatory models. Fizz1 was overexpressed in the lung prior to challenge with bleomycin or silica. Both models induce inflammatory changes leading to fibrosis; however the inflammatory responses are different with bleomycin resulting in an oxidative injury associated with neutrophil influx, while silica leads to macrophage-related activation and formation of inflammatory nodules. With both challenges, overexpression of Fizz1 did not alter the degree of lung remodeling. In the bleomycin injury, expression of Fizz1 did cause a greater loss of body weight. Although the mode of action is not known, these data further support the concept that lung epithelial cell produced Fizz1 has systemic effects consistent with effect on systemic recruitment of bone marrow derived cells. While the precise mode of activity is not known, Fizz1 has been shown to alter IL-13 induced chemokine levels, inhibit nerve growth factor neuronal survival, and cause bone marrow cell migration in a transwell assay through interactions with Bruton’s tyrosine kinase[42]. Thus Fizz1 may act directly or indirectly by influences on cytokine or growth factor induced regulation.

In spite of an increase in Fizz1 transcripts induced by bleomycin in the lung, the results of our study do not support a role for Fizz1 produced in the lung epithelium as a direct cause of lung fibrosis. In the present study, single instillations of bleomycin and silica were used which cause a slowly resolving inflammation leading to diffused fibrosis or nodular fibrosis. In hypoxic injury, the injury is continuous and leads to pulmonary hypertension. Fizz1 may act in a localized fashion to increase fibrotic responses around pulmonary arterioles during chronic hypoxia but not to increase granuloma formation around S. mansoni parasites or retained silica particles. In addition, the differences in the activation of immune pathways, cellular sites of Fizz1 production such as macrophages and eosinophils, and the amount of Fizz1 produced may be responsible for observed differences between studies. Lack of observable histological and hydroxyproline differences in bleomycin or silica-induced lung remodeling in Fizz1 overexpressing mice could be due to doses of bleomycin or silica. While the doses used in this study have been used in previous studies, the tested dose may mask or not be a sufficient dose to show Fizz1 effects. Alternatively, other members of the Fizz family may be involved in pulmonary fibrosis. When challenged with bleomycin, Fizz2 knockout mice show a decrease in the absolute number of total BAL cells, cytokine gene expression and extracellular matrix deposition than control mice [40].

## Conclusions

The findings in the current study indicated that epithelial overexpression of Fizz1 promoted the recruitment of bone marrow-derived dendritic cells into the lung, but did not directly induce fibrosis or alter inflammatory models of fibrosis.

## Abbreviations

FACS, Fluorescent activated cell sorting; FIZZ, Found in inflammatory zone; RELM, Resistin like molecule; AM, Alveolar macrophages; IM, Intersttial macrophages; Dox, Doxycycline; Ova, Ovalbumin; CCSP, Clara cell secretory protein; PCR, Polymerase chain reaction; IT, Intratracheal; IL, Interleukin.

## Competing interests

The authors declared that they have no competing interest.

## Authors’ contributions

SKM, TRK and WDH were responsible for experimental design, data analysis and manuscript preparation. SKM performed the bleomycin and silicosis challenges. RE performed RT-PCR for chemokines, FACS analysis and manuscript preparation. JAK generated the Fizz1 transgene and transgenic mice and KRD performed mouse husbandry, breeding and genotyping. SS and CD performed histological staining, mouse husbandry, breeding and biochemical studies. All authors read and approved the final manuscript

## Supplementary Material

Additional file 1 **Figure S1.** Fizz1 overexpression has no significant effect on CCL3 and CCL19 transcripts in the lung. CCSP/Fizz1 mice were fed with or without DOX for 5 days and transcripts for CCL3 and CCL19 were measured in the total lung transcripts using Real Time PCR. Figure S2. Fizz1 overexpression has no effect on bleomycin-induced increases in CD45 + CD11b + cells in the lung. Total lung cells were stained with anti-CD11b and anti-CD45 antibodies at day10 post bleomycin or saline. Gating strategy of CD45 + CD11b + cells in the lungs of bleomycin exposed and Fizz1 overexpressing mice compared to CCSP/- mice exposed to bleomycin or saline. Bleomycin treatment has increased the percentage of CD45 + CD11b + cells. Fizz1 overexpression had no modifying effects on bleomycin-induced increase in the percentage of CD45 + CD11b + cells. Click here for file
