# Supplementary material for: Resistin-like molecule alpha1 (Fizz1) recruits lung dendritic cells without causing pulmonary fibrosis
Source: Respir Res. 2012 Jun 22;13(1):51. doi: 10.1186/1465-9921-13-51 (PMC3485088; doi:10.1186/1465-9921-13-51)
Supplement: Additional file 1 — Figure S1. Fizz1 overexpression has no significant effect on CCL3 and CCL19 transcripts in the lung. CCSP/Fizz1 mice were fed with or without DOX for 5 days and transcripts for CCL3 and CCL19 were measured in the total lung transcripts using Real Time PCR. Figure S2. Fizz1 overexpression has no effect on bleomycin-induced increases in CD45 + CD11b + cells in the lung. Total lung cells were stained with anti-CD11b and anti-CD45 antibodies at day10 post bleomycin or saline. Gating strategy of CD45 + CD11b + cells in the lungs of bleomycin exposed and Fizz1 overexpressing mice compared to CCSP/- mice exposed to bleomycin or saline. Bleomycin treatment has increased the percentage of CD45 + CD11b + cells. Fizz1 overexpression had no modifying effects on bleomycin-induced increase in the percentage of CD45 + CD11b + cells. [file 1465-9921-13-51-S1.pdf]

Supplementary Data

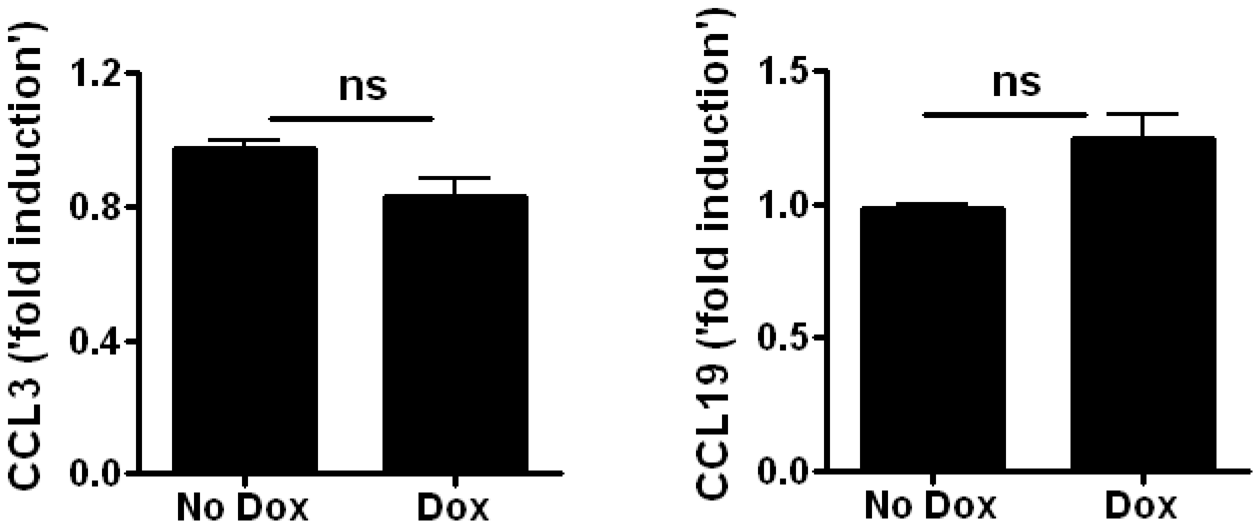

Supplementary Figure 1. Fizz1 overexpression has no significant effect on CCL3 and CCL19 transcripts in the lung. CCSP/Fizz1 mice were fed with or without DOX for 5 days and transcripts for CCL3 and CCL19 were measured in the total lung transcripts using Real Time PCR.

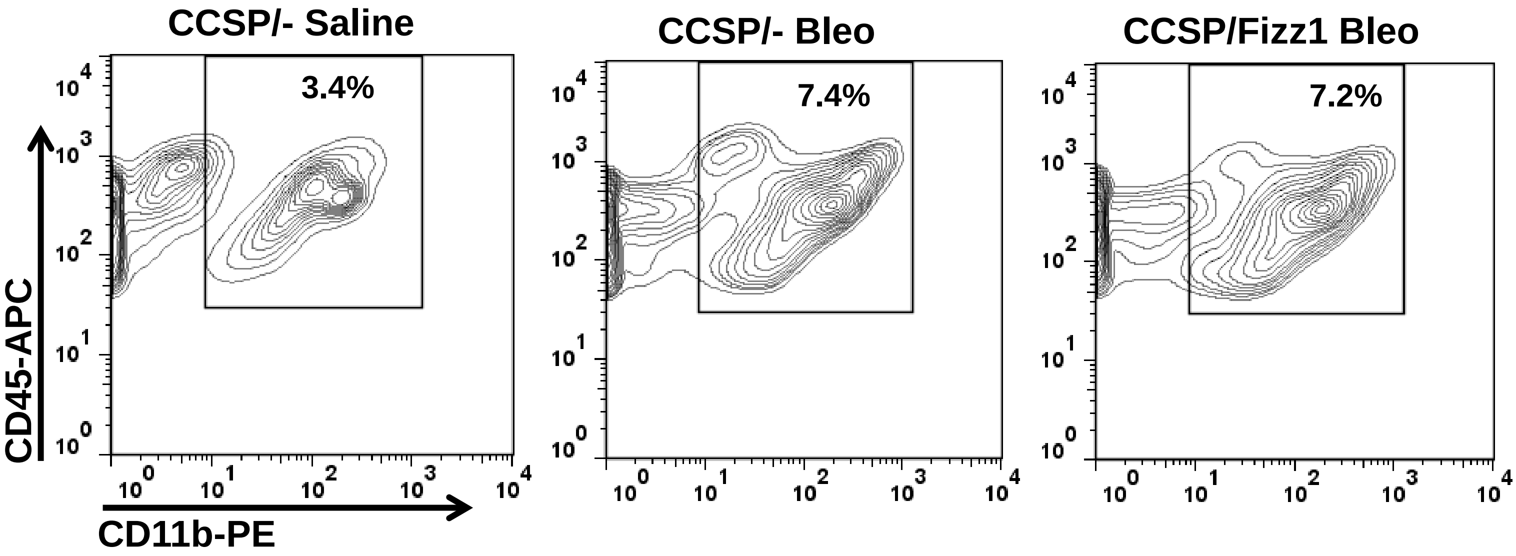

Supplementary Figure 2. Fizz1 overexpression has no effect on bleomycin-induced increases in CD45+CD11b+ cells in the lung. Total lung cells were stained with anti-CD11b and anti-CD45 antibodies at day10 post bleomycin or saline. Gating strategy of CD45+CD11b+ cells in the lungs of bleomycin exposed and Fizz1 overexpressing mice compared to CCSP/- mice exposed to bleomycin or saline. Bleomycin treatment has increased the percentage of CD45+CD11b+ cells. Fizz1 overexpression had no modifying effects on bleomycin-induced increase in the percentage of CD45+CD11b+ cells .
